# Karyotype description and comparative analysis in Ringed Kingfisher and Green Kingfisher (Coraciiformes, Alcedinidae)

**DOI:** 10.3897/CompCytogen.v12i2.23883

**Published:** 2018-05-10

**Authors:** Tiago Marafiga Degrandi, Jean Carlo Pedroso de Oliveira, Amanda de Araújo Soares, Mario Angel Ledesma, Iris Hass, Analía del Valle Garnero, Ricardo José Gunski

**Affiliations:** 1 Universidade Federal do Paraná, Av. Coronel Francisco Heráclito dos Santos, s/n, Curitiba, Paraná, Brazil; 2 Universidade Federal de Minas Gerais, Av. Pres. Antônio Carlos, 6627 – Pampulha, Belo Horizonte, Minas Gerais, Brazil; 3 Parque Ecológico El Puma – Candelaria, Misiones, Argentina; 4 Universidade Federal do Pampa, Rua Aluízio Barros Macedo, BR 290, km 423 Bairro Piraí, São Gabriel, Rio Grande do Sul, Brazil

**Keywords:** Aves, chromosome, evolution, karyotype

## Abstract

Kingfishers comprise about 115 species of the family Alcedinidae, and are an interesting group for cytogenetic studies, for they are among birds with most heterogeneous karyotypes. However, cytogenetics knowledge in Kingfishers is extremely limited. Thus, the aim of this study was to describe the karyotype structure of the Ringed Kingfisher (*Megaceryle torquata* Linnaeus, 1766) and Green Kingfisher (*Chloroceryle americana* Gmelin, 1788) and also compare them with related species in order to identify chromosomal rearrangements. The Ringed Kingfisher presented 2n = 84 and the Green Kingfisher had 2n = 94. The increase of the chromosome number in the Green Kingfisher possibly originated by centric fissions in macrochromosomes. In addition, karyotype comparisons in Alcedinidae show a heterogeneity in the size and morphology of macrochromosomes, and chromosome numbers ranging from 2n = 76 to 132. Thus, it is possible chromosomal fissions in macrochromosomes resulted in the increase of the diploid number, whereas chromosome fusions have originated the karyotypes with low diploid number.

## Introduction

Avian karyotypes are characterized by internal variation in the size of chromosomes, presenting two distinct groups, macrochromosomes and microchromosomes. About eight pairs of macrochromosomes are seen in most of birds, and the remaining are microchromosomes (Rodionov 1996). Diploid number also varies, including species with a low diploid number such as *Burhinus oedicnemus* Linnaeus, 1758 (Charadriiformes) 2n = 40 (Nie et al. 2009), and high 2n = 136-142 in *Corythaixoides concolor* Smith, 1833 (Musophagiformes) (Christidis 1990), but most of the species exhibit karyotypes with 2n = 74–86 (Tegelstrom and Ryttman 1981).

Studies of karyotype structure in birds have given valuable information about evolutionary relationships. Chromosome painting shows that, although relatively conserved, the macrochromosomes evolve through several intra and inter-chromosomal rearrangements (de Oliveira et al. 2010, Kretschmer et al. 2014). While Tandem fusions between microchromosomes and micro- with macrochromosomes have resulted in decrease of diploid number (Nishida et al. 2008, Nie et al. 2009, de Oliveira et al. 2010, 2013). Chromosome fission in recurrent breakpoints has been documented in macrochromosomes, and can result in increase of chromosome number (Skinner and Griffin 2012, Degrandi et al. 2017).

In relation to the sex chromosomes of birds, males have a homogametic ZZ pair and female have a heterogametic ZW (Schartl et al. 2015). The Z chromosome is a highly conserved macrochromosome and it comprises 7% of the haploid genome (Graves and Shetty 2001). In Piciformes, Bucerotiformes, and Coraciiformes the Z chromosome is often the largest chromosome of the complement (de Oliveira et al. 2017). Whereas the W chromosome is highly variable in size, and has been observed from homomorphic to Z in Paleognaths Ratite (Nishida-Umehara et al. 2007) to a small and heterochromatic with variable size in Neognaths birds (Graves and Shetty 2001). This size variation has been attributed to a differential accumulation and degradation of repetitive DNAs (de Oliveira et al. 2017). Also, a multiple sex chromosome system was recently described for the Adelie Penguin (*Pygoscelis adeliae* Hombron et Jacquinot, 1841/ Sphenisciformes) where males have Z_1_Z_1_Z_2_Z_2_ and females Z_1_Z_2_W (Gunski et al. 2017).

Kingfishers (Alcedinidae) comprises a diverse family of birds with approximately 115 species distributed worldwide (Gill and Donsker 2017). They are an interesting group for cytogenetic studies since they are among birds with most heterogeneous karyotypes. However, knowledge about cytogenetics in Kingfishers is extremely limited. There are records for *Dacelo novaeguineae* Hermann, 1783, 2n = 76, *Halcyon smyrnensis* Linnaeus, 1758, 2n = 76, *Halcyon pileata* Boddaert, 1783, 2n = 84, *Alcedo atthis* Linnaeus, 1758, 2n = 132, *Ceyx azureus* Latham, 1801, 2n = 122, and *Ceryle rudis* Linnaeus, 1758, 2n = 82 (De Boer and Belterman 1980, Xiaozhuang and Qingwei 1989, Christidis 1990, Youling et al. 1998, Garg and Shrivastava 2013).

The Ringed Kingfisher, *Megaceryle torquata* Linnaeus, 1766 and the Green Kingfisher, *Chloroceryle americana* Gmelin, 1788 belong to subfamily Cerylinae and their karyotypes are unknown (Moyle 2006). In view of this, the present study aimed to describe the karyotype structure of these species. Secondly, we sought to gather karyotype information from Alcedinidae in order to compare them and to identify the chromosomal rearrangements.

## Material and methods

### Samples and location

**Figure 1.**
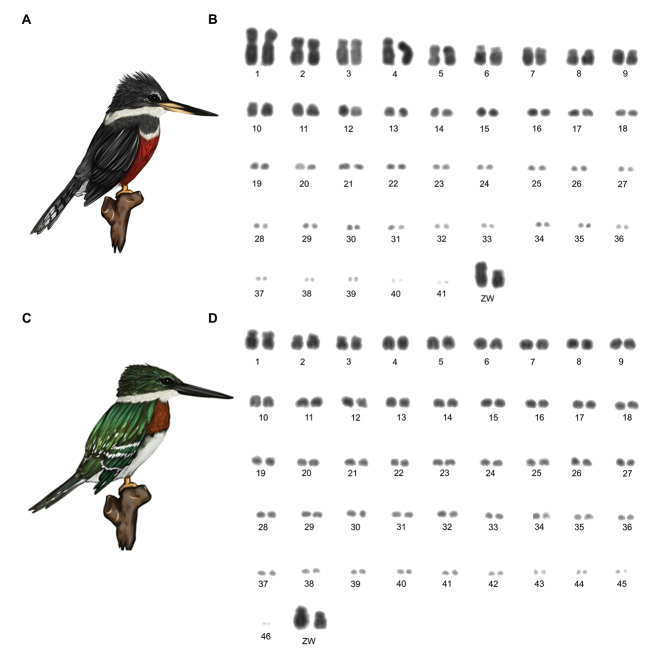
Ringed Kingfisher *Megaceryle torquata* **(A)**, and karyotype with 2n = 84 **(B)**. Green Kingfisher *Chloroceryle americana* (**C**), and karyotype with 2n = 94 **(D)**.

The karyotype of one male and one female of *Megaceryle torquata* (Fig. 1A) collected at the Parque Ecológico El Puma in Argentina, and two males and one female of *Chloroceryle americana* (Fig. 1C) from Santa Maria/Rio Grande do Sul, Brazil were analyzed for this work. Specimens were collected according to license SISBIO 44173-1 and animal research ethics committee (CEUA 018/2014).

### Cell culture

Mitotic chromosomes in *M. torquata* specimens were obtained by lymphocyte culture according to Moorhead et al. (1960). In short, blood samples were incubated in medium PBMax (Gibco) for 72 hours at 38 °C. In the last hour of incubation, 0,001 ml of colchicine solution (0.05%) was added. After these procedures, the cells were centrifuged and pellet was incubated in 10 mL of hypotonic solution (0.075 M KCl) for 20 min, followed by fixation in three washes with Methanol: Acetic acid 3:1 solution.

In *C. americana*, mitotic cells were obtained from bone marrow according to Garnero and Gunski (2000). Initially, bone marrow was extracted from femurs and incubated in a 10 ml of RPMI 1640 medium with 0,001 ml of colchicine solution (0.05%) at 39 °C for 1 hour. Finally, cells were incubated in 10 ml of hypotonic solution (0.075 M KCl) for 20 minutes. Then cells were washed three times with Methanol: Acetic acid 3:1 solution.

### Chromosomal analyses

The diploid number was determined by analyzing approximately 40 metaphases per specimen, by conventional 0,8% Giemsa staining solution. Karyotypes were organized according to chromosome size and differential staining CBG-banding (Sumner 1972) was applied to identify the W chromosome.

Morphometry of the first 15 autosomal chromosomes pairs and the ZW sex chromosomes, were performed in Alcedinidae species available. Centromeric index (CI) was estimated by ratio of short arm length by total chromosome length. Nomenclature for chromosome morphology were performed according to Guerra (1986) using CI index.

## Results

The Ringed Kingfisher presented chromosome number of 2n = 84 (Figure 1B). The chromosome set is composed of ten biarmed pairs, being the submetacentric pairs (1, 3 and 4), metacentric (2, 5, 8 and 13) and acrocentric (6, 7 and 9). The remaining autosomes are telocentric. Z and W are both submetacentric macrochromosomes, with size similar to chromosome 4 and 9, respectively.

The Green Kingfisher had a diploid number of 2n = 94 (Fig. 1D), consisting of only four biarmed pairs, where 1, 2 and 3 are submetacentric and 12 is metacentric. All the other chromosome pairs are telocentric. The Z chromosome is submetacentric and is the largest chromosome of the karyotype, while the W chromosome is submetacentric with size between 1 and 2.

**Figure 2.**
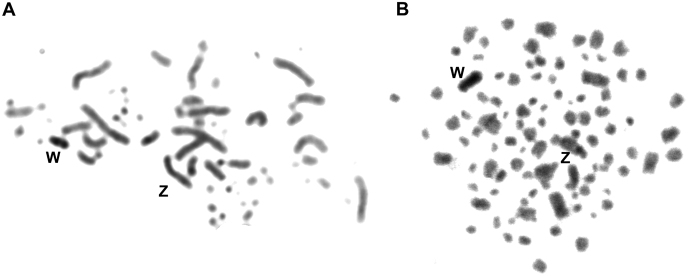
Comparative C-banding analysis of the Ringed Kingfisher *Megaceryle torquata* **(A)** and the Green Kingfisher *Chloroceryle americana* **(B)**.

C-banding analysis allowed correct identification of the W chromosome, since both species presented a highly heterochromatic pattern for this chromosome (Fig. 2A and B). The Z chromosome was euchromatic in both species. However, in *C. americana* a positive staining was observed near the centromere (Fig. 2 B).

In the literature, chromosome data were found for *C. rudis*, *H. pileata*, *A. atthis*, *H. smyrnensis*, *D. novaeguineae*, and *C. azureus* (Table 1). Unfortunately, for *H. smyrnensis*, *D. novaeguineae*, *C. azureus* only the diploid number was available. Despite this, some observations can be made: i) diploid number is highly variable; ii) number of biarmed chromosomes (metacentric, submetacentric, and acrocentric) was also variable; iii) the Z chromosome is a conserved submetacentric chromosome; and iv) the W chromosome morphology is variable among species, appearing as metacentric or submetacentric.

**Table 1.** Karyotype information’s in Alcedinidae species.

Species	2n	Nº biarmed	1	2	3	4	5	6	7	8	9	10	11	12	13	14	15	Z	W	Reference
*Chloroceryle americana*	94	4	S	S	S	T	T	T	T	T	T	T	T	M	T	T	T	S	S	Present work
*Ceryle rudis*	82	13	M	M	M	M	M	M	S	S	A	A	A	A	A	T	T	S	M	Garg and Shrivastava 2013.
*Megaceryle torquata*	84	10	S	M	S	S	M	A	A	S	A	T	T	T	M	T	T	S	S	Present work
*Halcyon pileata*	84	12	M	M	S	S	M	M	M	S	T	T	M	T	M	M	S	S	M	Xiaozhuang and Qingwei 1989.
*Halcyon smyrnensis*	76	–	–	–	–	–	–	–	–	–	–	–	–	–	–	–	–	–	–	Youling et al. 1998.
*Dacelo novaeguineae*	76	–	–	–	–	–	–	–	–	–	–	–	–	–	–	–	–	–	–	De Boer and Belterman 1980.
*Alcedo atthis*	132	15	M	M	M	S	M	M	M	M	S	M	S	M	M	M	M	S	M	Xiaozhuang and Qingwei 1989.
*Ceyx azureus*	122	–	–	–	–	–	–	–	–	–	–	–	–	–	–	–	–	–	–	Christidis 1990.

2n= diploid number; Nº biarmed= Number of biarmed autosomes; Chromosome morphology: (M=metacentric, S=submetacentric, A=Acrocentric and T=Telocentric); - = Not was possible to obtain the information in original work; Species names in accordance to IOC WORLD BIRD LIST (7.3) http://dx.doi.org/10.14344/IOC.ML.7.

## Discussion

Unfortunately, forty years after the publication of the karyotype of *D. novaguineae* (*D. gigas* by De Boer and Beltrman 1980), information about cytogenetics of Alcedinidae species is still limited. Nevertheless, comparisons done in this work (Tab. 1) show that Kingfishers present karyotype plasticity, evidenced by variation in diploid number, number of biarmed chromosomes, and in size and morphology of macrochromosomes.

According to White (1977), chromosome fusions result in the reduction of diploid number and increase of number of biarmed chromosomes, while chromosome fissions increase the diploid number and decrease the number of biarmed chromosomes. These mechanisms appear to be adequate to explain the differences in the karyotypes of Alcedinidae species.

In this work, the increasing of diploid number observed in *M. torquata* (2n = 84) to *C. americana* (2n = 94), (Fig. 1B and D) may have originated by chromosome fissions. Some characteristics support this hypothesis, such as, the number of biarmed chromosomes is reduced from 9 pairs in *M. torquata* for to 4 in *C. americana*, and Z chromosome size is similar to chromosome 4 in *M. torquata*, while in *C. americana*, the Z chromosome is the largest in the karyotype. However, experiments with chromosome painting with specific probes could confirm these hypotheses.

According to Graves and Shetty (2001) Z chromosome size is conserved in most birds. So, Z chromosome size in relation to other macrochromosomes can be considered as a marker for size and evidence of occurrence of chromosome fission or fusions. Chromosome W in *M. torquata* and *C. americana* did not present differences and shows a pattern of heterochromatinization, similar of what has been observed in other Neognaths species. However, when compared to other species of Kingfishers, it is observed that there is a variation in chromosome morphology, ranging from metacentric to submetacentric.

## Conclusion

Kingfishers present interesting chromosomal characteristics. These species have a diploid number which is highly variable and probably originated by fusions and/or fissions involving macrochromosomes. Hence rearrangements in macrochromosomes result in size and morphology variations, characterizing an intra-familial karyotypic heterogeneity. Absence of G-banding pattern and chromosome painting data did not allow comparisons. Therefore, we hope that this work may encourage the development of other cytogenetic studies in Kingfishers, and that our hypothesis of fission and chromosomal fusions as mechanisms responsible for karyotypes differentiation in Kingfishers can be confirmed.

## References

[B1] Christidis L (1990) Animal cytogenetics 4: Chordata 3 B: Aves. Gebrüder Borntraeger, Berlin, Germany, 55–57.

[B2] De Boer LEM Belterman RHR (1980) The karyotypes of two New Guinean birds: *Dacelo gigas* (Coraciiformes: Alcedinidae) and *Goura victoria* (Columbiformes: Columbidae). Chromosome Information Service 29: 17–18.

[B3] Degrandi TM Garnero ADV O’Brien PCM Ferguson-Smith MA Kretschmer R de Oliveira EHC Gunski RJ (2017) Chromosome painting in *Trogon* *s. surrucura* (Aves, Trogoniformes) reveals a karyotype derived by chromosomal fissions, fusion, and inversions. Cytogenetic and Genome Research 151: 208–215. https://doi.org/10.1159/000471782 28501862 10.1159/000471782

[B4] de Oliveira EHC Tagliarini MM Rissino JD Pieczarka JC Nagamachi CY O’Brien PCM Ferguson-Smith MA (2010) Reciprocal chromosome painting between white hawk (*Leucopternis albicollis*) and chicken reveals extensive fusions and fissions during karyotype evolution of Accipitridae (Aves, Falconiformes). Chromosome Research 18: 349–355. https://doi.org/10.1007/s10577-010-9117-z 20198417 10.1007/s10577-010-9117-z

[B5] de Oliveira EHC Tagliarini MM dos Santos MS O’Brien PCM Ferguson-Smith MA (2013) Chromosome painting in three species of Buteoninae: A cytogenetic signature reinforces the monophyly of south American species. PLoS ONE 8(7): e70071. https://doi.org/10.1371/journal.pone.0070071 10.1371/journal.pone.0070071 PMC3724671 23922908

[B6] de Oliveira TD Kretschmer R Bertocchi NA Degrandi TM de Oliveira EHC Cioffi MDB Garnero ADV Gunski RJ (2017) Genomic Organization of Repetitive DNA in Woodpeckers (Aves, Piciformes): Implications for Karyotype and ZW Sex Chromosome Differentiation. PLoS ONE 12(1): e0169987. https://doi.org/10.1371/journal.pone.0169987 10.1371/journal.pone.0169987 PMC5230766 28081238

[B7] Garnero ADV Gunski RJ (2000) Comparative analysis of the karyotypes of *Nothura maculosa* and *Rynchotus rufescens* (Aves: Tinamidae). A case of chromosomal polymorphism. The Nucleus 43: 64–70.

[B8] Garg HK Shrivastava (2013) A Genetic Surveillance of Kingfisher and Bee Eater. European Journal of Biotechnology and Bioscience 1(2): 1–5. http://www.biosciencejournals.com/vol1/issue2/pdf/23.1.pdf

[B9] Gill F Donsker D (2017) IOC World Bird List. V.7.1 http://www.worldbirdnames.org/ [Accessed 18. October 2017] https://doi.org/10.14344/IOC.ML.7.1

[B10] Graves JAM Shetty S (2001) Sex from W to Z: Evolution of vertebrate sex chromosomes and sex determining genes. Journal of Experimental Zoology 290: 449–462. https://doi.org/10.1002/jez.1088 11555852 10.1002/jez.1088

[B11] Guerra MS (1986) Reviewing the chromosome nomenclature of Levan et al. Revista Brasileira de Genética 4: 741–743.

[B12] Gunski RJ Cañedo AD Garnero ADV Ledesma MA Coria N Montalti D Degrandi TM (2017) Multiple sex chromosome system in penguins (*Pygoscelis*, Spheniscidae). Comparative Cytogenetics 11(3): 541–552. https://doi.org/10.3897/CompCytogen.v11i3.13795 29093802 10.3897/CompCytogen.v11i3.13795 PMC5646662

[B13] Kretschmer R Gunski RJ Garnero ADV Furo Ido O’Brien PCM Ferguson-Smith MA de Oliveira EHC (2014) Molecular cytogenetic characterization of multiple intrachromosomal rearrangements in two representatives of the genus *Turdus* (Turdidae, Passeriformes). PLoS ONE 9(7): e103338. https://doi.org/10.1371/journal.pone.0103338 10.1371/journal.pone.0103338 PMC4110018 25058578

[B14] Moorhead RS Howell PC Mellman WJ Batteps DM Hundgerford DA (1960) Chromosomes preparations of leukocytes cultured from human peripheral blood. Experimental Cell Research 2: 613–616. https://doi.org/10.1016/0014-4827(60)90138-5 10.1016/0014-4827(60)90138-5 13772379

[B15] Moyle RG (2006) Molecular phylogeny of Kingfishers (Alcedinidae) with insights into early biogeographic history. The Auk 123(2): 487–499. https://doi.org/10.1642/0004-8038(2006)123[487:AMPOKA]2.0.CO;2

[B16] Nie W O’Brien PCM Ng BL Fu B Volobouev V Carter NP Ferguson-Smith MA Yang F (2009) Avian comparative genomics: reciprocal chromosome painting between domestic chicken (*Gallus gallus*) and the stone curlew (*Burhinus oedicnemus*, Charadriiformes)—An atypical species with low diploid number. Chromosome Research 17(1): 99–113. https://doi.org/10.1007/s10577-009-9021-6 19172404 10.1007/s10577-009-9021-6 PMC2697597

[B17] Nishida C Ishijima J Kosaka A Tanabe H Habermann FA Griffin DK Matsuda Y (2008) Characterization of chromosome structures of Falconinae (Falconidae, Falconiformes, Aves) by chromosome painting and delineation of chromosome rearrangements during their differentiation. Chromosome Research 16: 171–181. https://doi.org/10.1007/s10577-007-1210-6 18293111 10.1007/s10577-007-1210-6

[B18] Nishida-Umehara C Tsuda Y Ishijima J Ando J Fujiwara A Matsuda Y Griffin DK (2007) The molecular basis of chromosome orthologies and sex chromosomal differentiation in palaeognathous birds. Chromosome Research 15: 721–734. https://doi.org/10.1007/s10577-007-1157-7 17605112 10.1007/s10577-007-1157-7

[B19] Rodionov AV (1996) Micro vs. macro: a review of structure and functions of avian micro- and macrochromosomes. Russian Journal of Genetics 32(5): 517–527.

[B20] Schartl M Schmid M Nanda I (2015) Dynamics of vertebrate sex chromosome evolution: from equal size to giants and dwarfs. Chromosoma 125: 553–571. https://doi.org/10.1007/s00412-015-0569-y 26715206 10.1007/s00412-015-0569-y

[B21] Skinner BM Griffin DK (2012) Intrachromosomal rearrangements in avian genome evolution: evidence for regions prone to breakpoints. Heredity 108: 37–41. https://doi.org/10.1038/hdy.2011.99 22045382 10.1038/hdy.2011.99 PMC3238122

[B22] Sumner AT (1972) A simple technique for demostrating centromeric heterocrhomatin. Experimental cell research 75: 304–306. https://doi.org/10.1016/0014-4827(72)90558-7 4117921 10.1016/0014-4827(72)90558-7

[B23] Tegelstrom H Ryttman H (1981) Chromosomes in birds (Aves): evolutionary implications of macro- and microchromosome numbers and lengths. Hereditas 94: 225–233. https://doi.org/10.1111/j.1601-5223.1981.tb01757.x

[B24] Xiaozhuang B Qingwei L (1989) Studies on the karyotypes of birds V. The 20 species of climber birds (Aves). Zoological Research 10(4): 309–317. http://www.zoores.ac.cn/CN/Y1989/V10/I4/309

[B25] White MJD (1977) Os cromossomos. Editora Nacional, EDUSP, São Paulo, 196 pp.

[B26] Youling C Qiujin Z Xiaoyin H Zhaohe T (1998) Comparative studies on karyotype of 5 species of climber birds. Wuyi Science Journal 14: 218–221.

